# Correction: Autism Screening Using Parent’s Observations of Social Interactions (POSI) in High-Risk Infants

**DOI:** 10.1007/s10803-026-07320-5

**Published:** 2026-05-06

**Authors:** Lydia Vielmetti, Aarabhi Rajagopal, Robin Manus, Sharon Veis, Raye-Ann deRegnier

**Affiliations:** 1 Department of Pediatrics, Division of Neonatology, Lurie Children’s Hospital, 225 East Chicago Avenue, Chicago, IL 60611 USA; 2 Department of Pediatrics, Division of Hospital Medicine, Seattle Children’s Hospital, Seattle, WA USA

**Correction to: Journal of Autism and Developmental Disorders**

10.1007/s10803-026-07239-x

In this article Fig. 3 appeared incorrectly and has now been corrected. For completeness and transparency, the old incorrect version and corrected version are displayed below.

Incorrected Fig. 3.


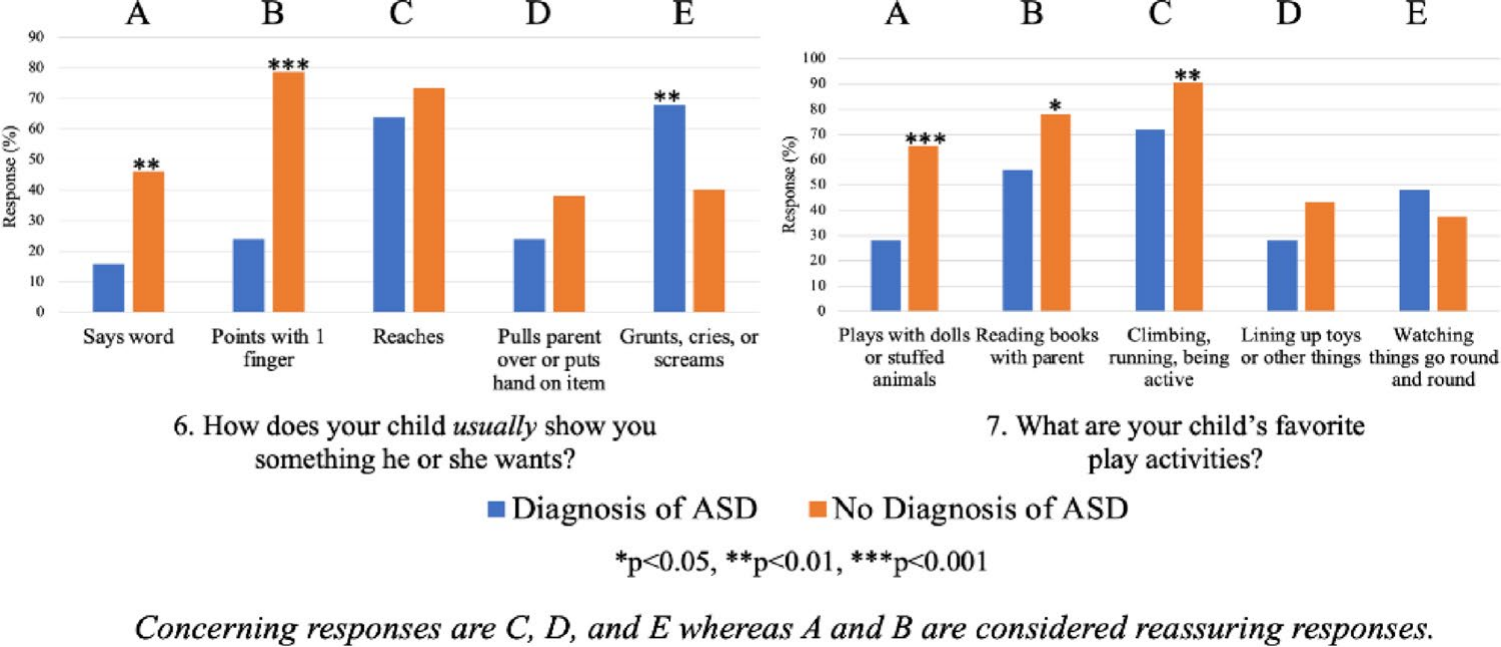


Corrected Fig. 3.

**Fig. 3:**
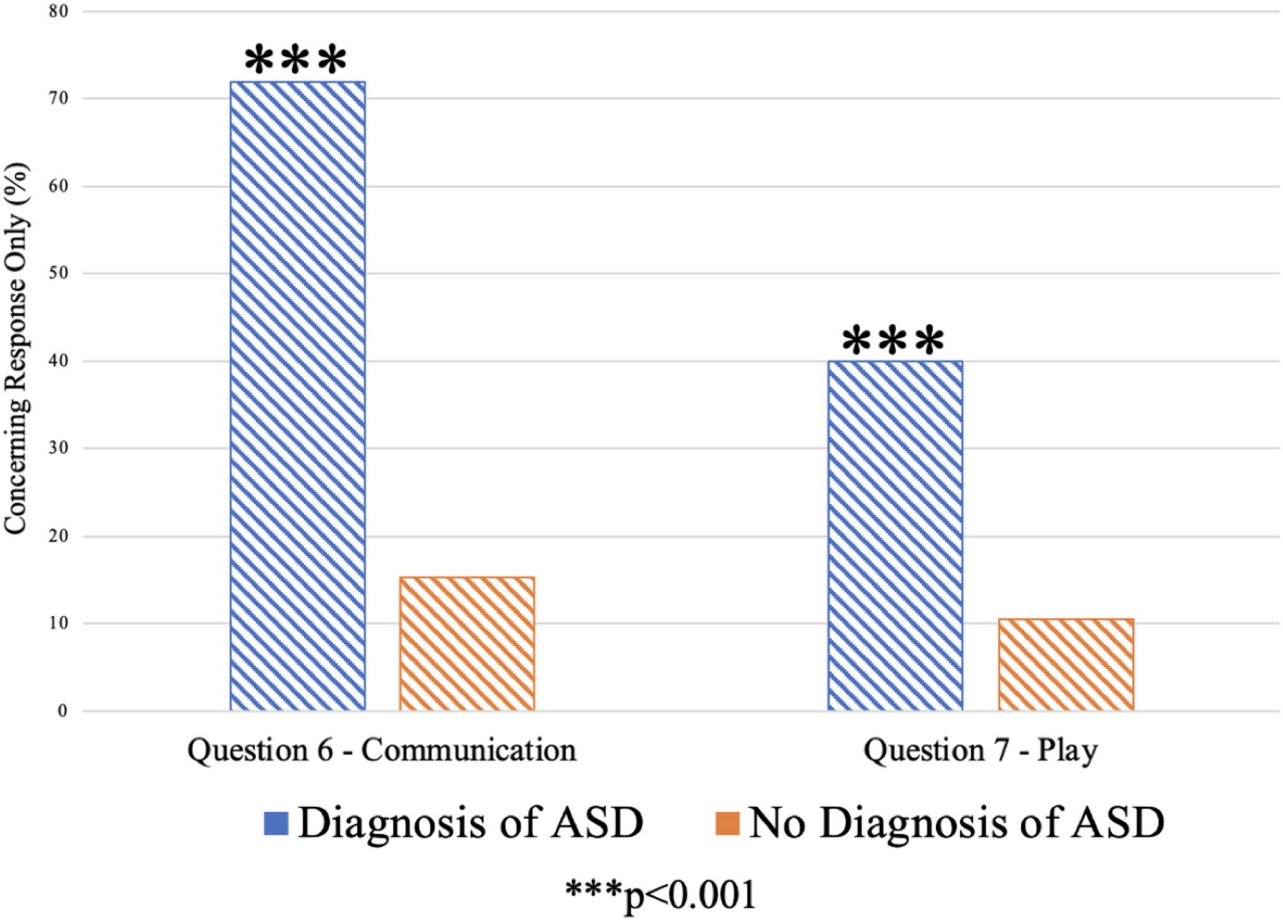
Proportion of children with only concerning responses to POSI questions 6 and 7 for children with diagnoses of ASD compared to children with no ASD diagnosis

The original article has been corrected.

